# Ground‐Truthing of MaxEnt Models Reveals Poor Predictive Accuracy for Lizards in the Mackenzie Basin, New Zealand

**DOI:** 10.1002/ece3.72155

**Published:** 2025-09-11

**Authors:** Scott D. Bourke, Steph M. Bennington, Sam Turner, Joanne M. Monks

**Affiliations:** ^1^ Department of Zoology University of Otago, Ōtākou Whakaihu Waka Dunedin New Zealand; ^2^ Department of Marine Science University of Otago, Ōtākou Whakaihu Waka Dunedin New Zealand; ^3^ Department of Conservation Te Papa Atawhai Twizel New Zealand

**Keywords:** gecko, herpetofauna, model validation, skink, Species Distribution Models

## Abstract

The potential utility of Species Distribution Models (SDMs) in conservation is apparent. One application for rare or highly cryptic taxa is using model predictions to increase the efficiency of sampling effort. Though this method is potentially powerful, the accuracy of model predictions is rarely tested in the field. Further, uncertainty remains about whether validation statistics reflect true model performance, particularly for species of high conservation concern. We assessed the usefulness of SDMs for predicting the distribution of six species of lizards in the Mackenzie Basin (Te Manahuna), Aotearoa New Zealand (NZ). We built MaxEnt models using readily available occurrence data and a publicly available suite of environmental predictors. We validated model performance using both data partitioning and independent occurrence records collected in the 2022/23 austral summer. Cross‐validation suggested that the top models for each species generated reasonably accurate predictions; however, for common species, predictive accuracy decreased notably when validating with independent data. Models for rare species performed more variably when validated with independent data; however, these models were overfit and based on few data, making it difficult to have confidence in the resulting abstractions. We suggest that limitations in historical occurrence data, current knowledge of species ecology and low resolution of predictor data likely restrict the relevance of predictive modelling for NZ lizard species. Whilst attractive to species managers and easy to generate, predictive models should be subject to ground‐truthing with temporally relevant data prior to being used to inform sampling effort.

## Graphical abstract

We ground‐truthed MaxEnt Species Distribution Models for six species of New Zealand lizards. Cross‐validation suggested that top models for each species generated reasonably accurate predictions; however, predictive accuracy was low for common species when validating with independent data. Whilst attractive to species managers and easy to generate, models should be ground‐truthed with temporally relevant data prior to their use in informing species management of sampling effort.

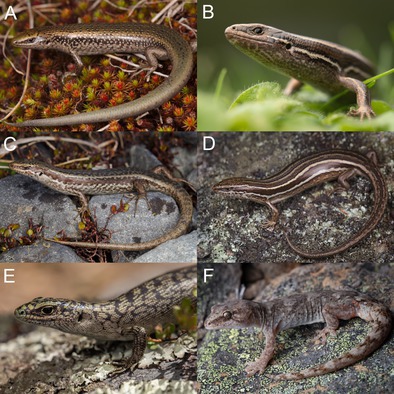

## 1 Introduction

Species Distribution Models (SDMs) provide a quantitative approach to understanding species' habitat use and range from simple associations with environmental features (e.g., Breckenridge 1956; Grinnell 1904) to more complex computational processes (e.g., Elith and Leathwick 2009; Le Lay et al. 2010). Modelled relationships can be used to predict distributions across space and time, providing a method to assess how species may respond to different climate regimes (e.g., Jarvie et al. 2022) or with changing threats (e.g., Brotons et al. 2004; Soberón et al. 2001). These abstractions have a variety of uses in conservation planning, such as to assess the suitability of translocation habitat (e.g., Jarvie et al. 2021), understand critical distributions of species (e.g., biodiversity hotspots; Volis and Tojibaev 2021), prioritise areas for spatial protection and legislation (Addison et al. 2013; Guisan et al. 2013; Volis and Tojibaev 2021) and predict the impact of biological invasions (Guisan et al. 2013; Soberón et al. 2001). Though touted as widely applicable, instances of managers using SDMs to inform decision making remain relatively rare (Cayuela et al. 2009; Guisan et al. 2013). Furthermore, if SDMs are to be valuable as a conservation tool, the accuracy of predictions is of paramount importance, and efforts are required to ensure that models accurately reflect distributions within the areas where they are being used.

Building accurate models is contingent on several factors, including the availability of robust occurrence data, knowledge of key drivers of distribution and having access to covariates on relevant scales. Many historical data repositories contain occurrence records that were collected using diverse methodologies and for different purposes (Newbold 2010). As such, they likely contain a number of biases and may not be robust to statistical testing (Aubry et al. 2017; Cayuela et al. 2009). Furthermore, many SDMs use publicly available covariates obtained from satellite records representing large spatial scales and taking long‐term averages (e.g., 100 m^2^; McCarthy et al. 2021). Coarse predictor variables may be less spatially or temporally relevant to the target species, and the resulting modelled relationships may poorly reflect a species' true distribution (Fei and Yu 2016; Jarnevich et al. 2018; Rondinini et al. 2006; Seaborn et al. 2021; Sor et al. 2017; Syfert et al. 2013). Even when drivers of distribution are well understood (e.g., Norbury et al. 2023), important covariates may be difficult to represent in SDMs (Rondinini et al. 2006). For example, intra‐ and interspecific interactions are often difficult to quantify and so are frequently omitted; models without these covariates may perform comparatively worse (e.g., Bennington et al. 2020; Jarnevich et al. 2018; Kass et al. 2020; Seaborn et al. 2021). Such issues are often exacerbated for highly endangered, naturally uncommon or newly discovered species, for which occurrence data are further limited (Pulliam and Babbitt 1997).

Due to the prevalence and varied uses of SDMs in conservation efforts, it is important that practitioners have a robust way to determine how well a model reflects true distribution. Commonly, this process involves using the model in question to predict response values for a portion of occurrence data withheld from model building (Chatfield 1995; Fielding and Bell 1997). The predictions can then be compared with the actual value of the withheld data, that is, presence or absence, to determine how frequently the model is correct. The ability of such a test to accurately assess model performance relies on obtaining validation data that is independent from the model building process. Thus, all occurrence data, both for model building and validation, must be collected in a statistically robust fashion, which is representative of a species' true distribution (Fei and Yu 2016; Phillips et al. 2009). These criteria are often not met, particularly for naturally rare or cryptic species (Aubry et al. 2017; Cayuela et al. 2009). If, instead, data with unknown biases are used to assess predictive performance, validation statistics may present a spurious estimation of a model's predictive ability and leave species managers uncertain as to whether their predictions are useful (Lahoz‐Monfort et al. 2014; Syfert et al. 2013).

For lizards, SDMs have been used to assess the impact of existing spatial protection (Corbalán et al. 2011), biological invasions (Jarnevich et al. 2018) and changing threats (Ballesteros‐Barrera et al. 2007) and predict the suitability of translocation habitat (Bellis et al. 2020; Jarvie et al. 2021). A promising, although rarely practiced, application of SDMs in this group is improved targeting of survey efforts (Fois et al. 2018; Guisan and Thuiller 2005; Guisan et al. 2006), which can be highly inefficient (Bell 2009; Hoare et al. 2013) and resource intensive (e.g., Bell and Patterson 2008). Increased sampling efficiency may be particularly beneficial for species for which extensive sampling effort has yielded little success (e.g., Sinbad skinks *O. pikitanga*, Bell and Patterson 2008; Monks et al. 2022) or in cases where finding new populations is the simplest way to improve species outlooks. In other taxa, predictive models have proven useful for refining search areas. For example, Le Lay et al. (2010) used ensemble modelling to define areas of high habitat suitability for rare plant species in the Swiss Alps. Subsequent sampling in these areas resulted in improved rates of detection compared to random sampling. Such approaches for lizard taxa could potentially be used to better target effort. Lizard species in Aotearoa New Zealand (NZ) are highly threatened, with 96% classed as ‘Threatened’ or ‘At Risk’ under the most recent threat classification (Hitchmough et al. 2021). Conservation efforts are hampered by a lack of up‐to‐date data on the distribution of species and limited funding (Böhm et al. 2013; Hitchmough et al. 2016, 2021; Towns et al. 2016). The most comprehensive repository of occurrence data for NZ lizards is the Department of Conservation's Herpetofauna database, which contains data from targeted surveys, historical sightings and incidental encounters.

These data could be used to generate predictive models for lizard species (e.g., Jarvie et al. 2022), which could be applied to improve the efficiency of sampling and, ultimately, improve the understanding of species' distributions. However, data were collected using a variety of methodologies and for diverse purposes. To ensure that model predictions derived from these data reflect reality, it is important to test outputs against contemporary distributions, i.e., ground‐truth the models.

Here, we aimed to assess the accuracy of SDMs built with publicly available data for lizards that occur within Te Manahuna/the Mackenzie Basin, a unique dryland system in NZ. We built correlative MaxEnt models for six lizard species using occurrence data from the DOC Herpetofauna database and using a publicly available suite of environmental predictors, the NZ Environmental Data Stack (NZEnvDS; McCarthy et al. 2021). Models were validated using (1) withheld occurrence data and (2) contemporary, independently collected distribution data from standardised survey methods. We compared validation statistics between methods to provide insight into the usefulness of model predictions for lizards in the Mackenzie Basin.

## 2 Methods

### 2.1 Study Species

**FIGURE 1:**
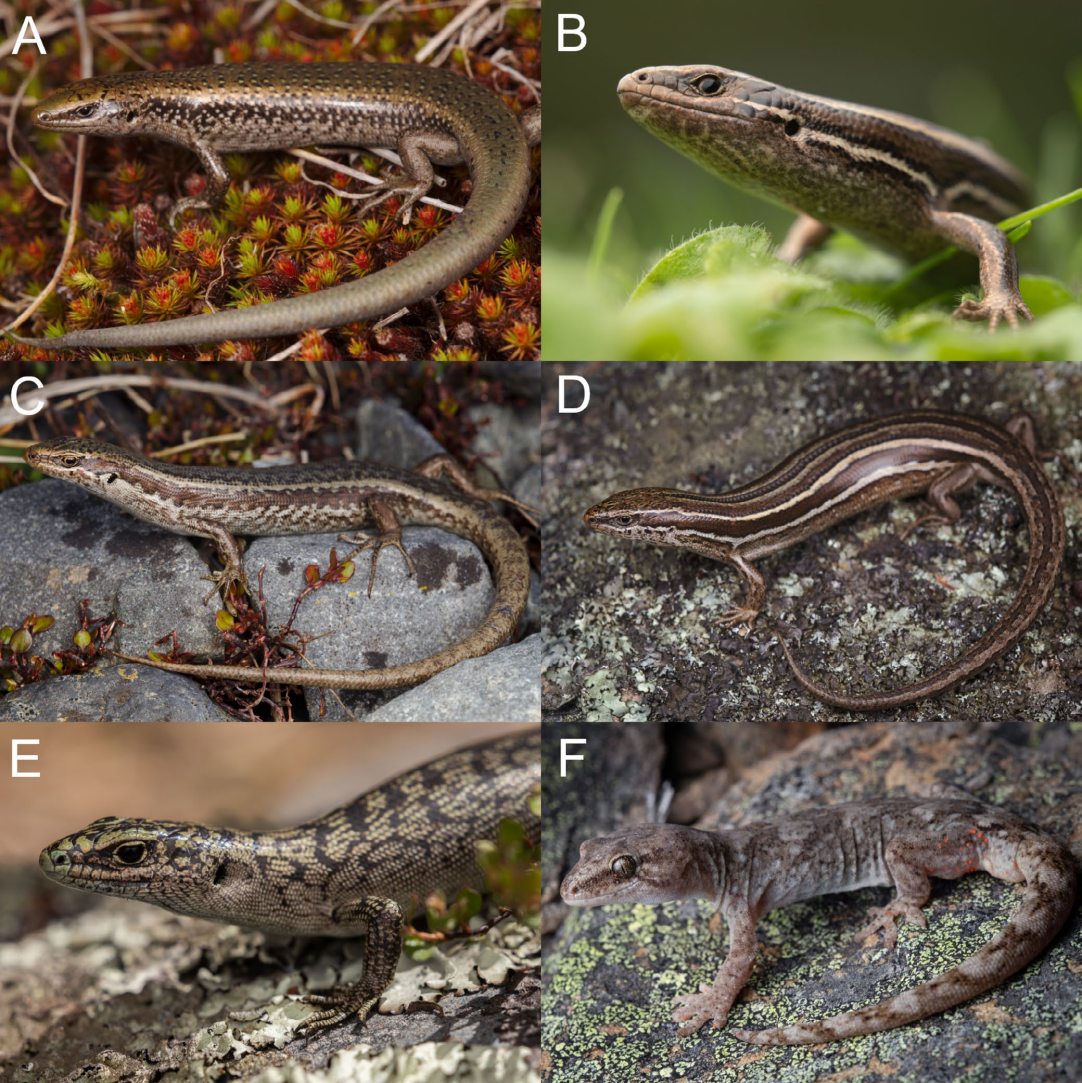
Photographs of the study species: (A) Mackenzie skink/*O. prasinum*, (B) Grass skink/*Oligosoma* aff. *polychroma* Clade 5, (C) Roamatimati skink/*O*. aff. *longipes* ‘southern’, (D) McCann's skink/*O. maccanni*, (E) Scree skink/*O. waimatense* and (F) Southern Alps gecko/*Woodworthia* ‘Southern Alps’. Photos: Samuel Purdie.

We built SDMs for five skink and one gecko species (Table 1 and Figure 1), including three common species, grass skink (*O*. aff. *polychroma* Clade 5), McCann's skink (*O. maccani*) and Southern Alps gecko (*Woodworthia* ‘Southern Alps’), and three rare species, including Mackenzie skink (*O. prasinum*), roamatimati skink (O. aff. longipes ‘southern’) and scree skink (*O. waimatense*).

**TABLE 1:** A summary of data used to construct correlative MaxEnt Species Distribution Models and the independent testing data. For each species, the number of occurrences present in the New Zealand's Department of Conservation Herpetofauna Database are given (*n*), as well as, those removed during modelling (Rem) and the resulting number used in modelling (Mod). The amount of independent testing data for all species including both presences (Pre) and absences (Abs), collected using trapping methods in the Mackenzie Basin during the 2022/23 summer. Threat statuses for all species are also provided, as per Hitchmough et al. 2021.

Species		Herpetofauna database			Independent data	
Common name/scientific name	Threat status	*n*	Rem	Mod	Pre	Abs
Grass skink/*Oligosoma* aff. *polychroma* Clade 5	Declining	891	262	629	138	1910
Mackenzie skink/*O. prasinum*	Nationally vulnerable	45	7	38	8	2040
McCann's skink/*O. maccanni*	Not threatened	1624	150	1474	369	1679
Roamatimati skink/*O*. aff. *longipes* ‘southern’	Declining	89	30	59	113	1935
Scree skink/*O. waimatense*	Nationally vulnerable	111	23	88	4	2044
Southern Alps gecko/*Woodworthia* ‘Southern Alps’	Not threatened	1077	114	963	96	1952

### 2.2 Study Site

**FIGURE 2:**
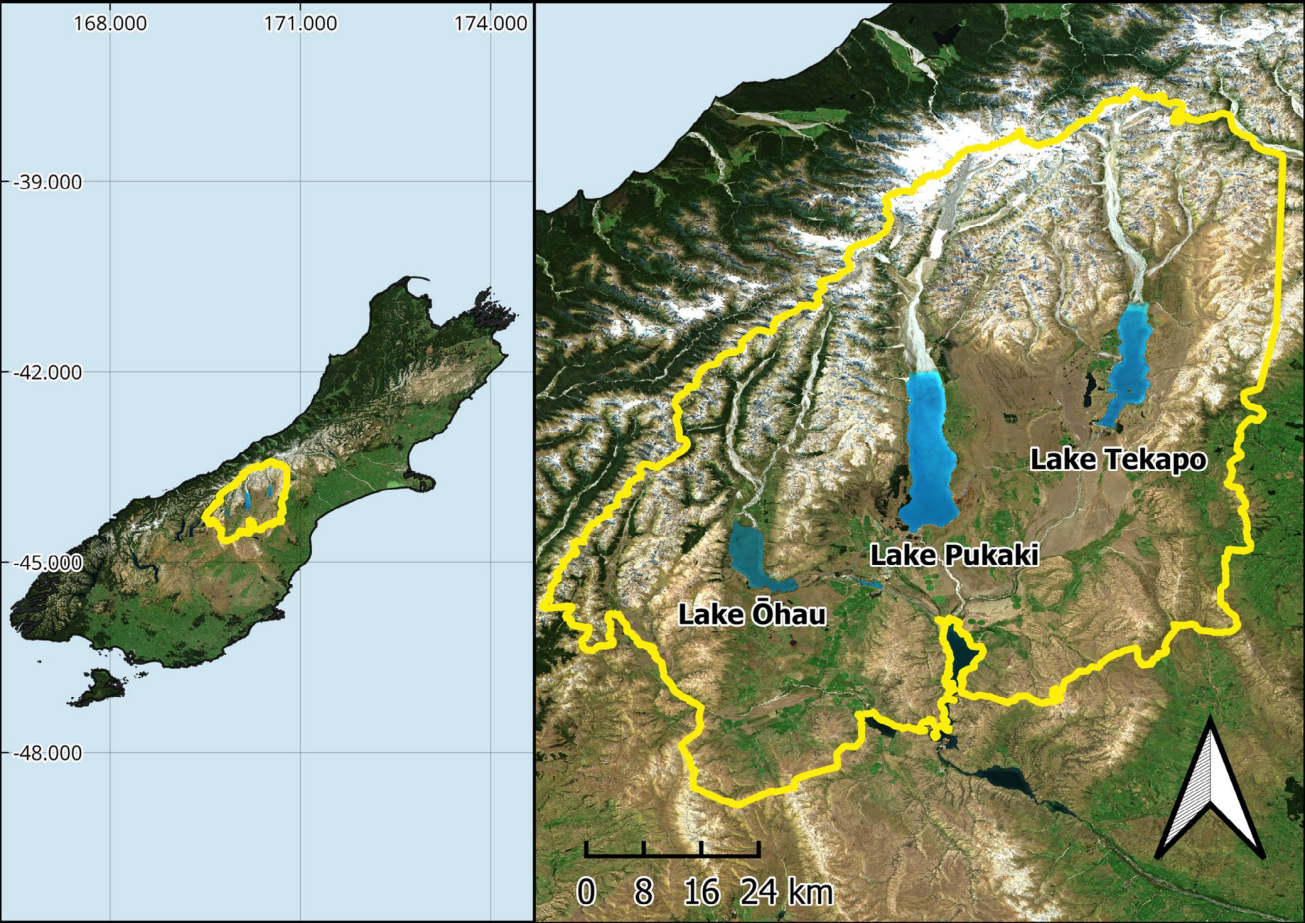
Map showing the position of the Mackenzie Basin in Aotearoa New Zealand (left) and the topography of the area, including the major lakes in the area. Satellite photography sourced from the Land Information New Zealand at 10 m resolution (2023–2024).

The Mackenzie Basin is an intermontane basin central to the South Island of NZ, flanked by the Southern Alps on the west and a series of lower ranges to the east (Figure 2). The basin is bisected by three major braided river systems (Pukaki, Tekapo and Ōhau) that have been significantly altered by local hydroelectric schemes (Abbott et al. 2019). These geographic features have resulted in a diversity of rare ecosystems on the basin floor, including moraines, outwash plains, glacial lakes and braided rivers (Caruso 2006; Williams et al. 2007; Wiser et al. 2013). Anthropogenic fire regimes cleared the basin of the majority of its original woody vegetation, leaving predominantly tussock grasslands interspersed with pockets of native scrub (McGlone 2001; Rogers et al. 2007). These landscapes support a high diversity of lizards with at least nine species known from the region (Purdie 2022; van Winkel et al. 2020).

### 2.3 Data Collection

SDMs were built using occurrence data from the DOC Herpetofauna Database, the largest collection of distribution data for NZ's lizard species (Table 1). The Herpetofauna Database is a curated collection of occurrence records that have been made since the 1900s, including sightings, live captures, dead recoveries, museum specimens and fossil records.

Occurrence records from six species known to occur in the Mackenzie Basin were selected from the database (Table 1). Three other species from the area (Lakes skink; *O*. aff. *chloronton* ‘West Otago’, cryptic skink; *O. inconspicuum* , jewelled gecko; *Nautilinus gemmeus*) were not included in subsequent analysis as no captures/sightings of these species were made when collecting independent testing data. We restricted the dataset to include only live occurrence records, excluding all museum specimens which often have far less accurate positional information. Locations of data were checked for feasibility, so occurrences outside of the species known range or within bodies of water (e.g., lakes or the sea) were excluded.

### 2.4 Predictor Variables

Models were built using seven predictor variables, including aspect (1 North to −1 South), land use (categorical with 17 levels), average annual precipitation, slope (degrees), annual solar radiation (hours), minimum temperature of the coolest month and maximum temperature of the warmest month. All variables except land use were taken from the NZEnvDS at their native resolution of 100 × 100 m (McCarthy et al. 2021). Land use was based on the New Zealand Landcover Database v5.0 shapefiles (Thompson et al. 2020). Shapefiles were rasterised with QGIS's ‘rasterize’ function, using the most up to date categories (‘2018 code’) to populate the raster (‘burn‐in’; QGIS Development Team 2023). Land use raster size was set to 100 m to match the resolution of the NZEnvDS rasters (McCarthy et al. 2021). All continuous variables were checked for collinearity using tables provided in McCarthy et al. (2021). All variables had pairwise collinearity values below 0.7 (Dormann et al. 2013). To control overfitting, we reduced the number of levels of the land use variable. We collapsed levels that were similar, i.e., Estuarine Open Water, River, Lake or Pond, or likely had a similar impact on lizards presence or absence, i.e., Exotic forest, Indigenous Forest, Broadleaved Indigenous Hardwoods and Forest‐Harvested (Appendix 1).

All predictor variables are included on the basis that they have a realistic ecological relationship with the presence/absence of lizards. Aspect (northness) and annual solar radiation are included as they both describe the availability of sunlight. Where sunlight is more available, heliothermic organisms have greater opportunity to optimally thermoregulate and therefore may prefer areas with greater sunlight availability (Reynolds and Casterlin 1979). This is especially relevant at temperate latitudes where ambient temperatures usually fall well below preferred body temperatures (Chukwuka et al. 2021; Virens and Cree 2019). Though likely correlated with altitude at extreme values, slope serves as a proxy for interstitial space and vegetation. Slope may be a particularly important predictor for species that can use scree slopes as primary habitat, including roamatimati (*O*. aff. *longipes* ‘Southern’) and scree skinks (*O. waimatense*) and Southern Alps geckos (*Woodworthia* ‘Southern Alps’; Purdie 2022; van Winkel et al. 2020). Both categorical variables were included to capture any general relationships that the assessed species have with habitat composition, exploring physical characteristics and vegetation/land management. Minimum temperature of the coldest month and maximum temperature of the warmest month were included to describe the lower and upper physiological boundaries of lizards (Hare and Cree 2016). Finally, annual precipitation is included on the basis that it may describe lizards' susceptibility to desiccation, important for NZ's lizards which may be particularly prone to high rates of cutaneous water loss (Hare and Cree 2016).

### 2.5 Model Building

The Herpetofauna Database contains only presence data. As such, we chose to build models using the maximum entropy (MaxEnt) algorithm, a method designed to take advantage of the power of such historical datasets (Elith et al. 2011; Phillips et al. 2006). MaxEnt models have robust predictive ability when compared with other model families and have been used for a wide array of modelling applications (Elith et al. 2006, 2011; Valavi et al. 2022; Virgili et al. 2017).

To create MaxEnt models, we used the ‘ENMevaluate’ function of the ‘ENMeval’ package (Kass et al. 2021) in the R environment (version 4.2.2). ENMeval allows parallel evaluation and tuning of SDMs with different feature classes and regularisation multipliers. Models tuned in this way consistently outperform those built with the default settings of MaxEnt (Kass et al. 2021). In this work, we avoid using complex combinations of predictor–response relationships to limit over‐parameterisation (Merow et al. 2013, 2014). As such, only models with linear, quadratic and linear‐quadratic feature classes were included.

Data were removed during modelling when they fell outside of raster data for any of the predictor variables or, more frequently, when they fell within the same raster cell as another occurrence. For Mackenzie skinks (*O. prasinum*) and roamatimati skinks, few occurrences were available in the Herpetofauna Database (*n* = 27 and *n* = 51, respectively). The paucity of occurrence data for these species may result in models that fail to aptly describe the full range of important habitat, highlighting a problem for rare or highly threatened species. To partially remedy this, we chose to include occurrence records that were within the study area (defined below) from *O. lineoocellatum* (*n* = 18) for Mackenzie skinks and *O. longipes* occurrences (*n* = 37) for roamatimati skinks, two closely related species (Melzer et al. 2017; Patterson 1997). These inclusions remedied issues where validation statistics were incomplete for certain models. Inclusions had little impact on model performance (both cross‐validation and to independent data) for either species, though they did decrease overfitting drastically for Mackenzie skink models.

### 2.6 Buffer Size and Background Points

The environmental range of data, for both occurrence and pseudo‐absence data, has large impacts on the shape and size of variable effects, especially at extremes (Thuiller et al. 2004; van Der Wal et al. 2009). The study area was defined by creating a 50 km buffer around all occurrence data. This distance was chosen as it created mostly contiguous study areas. For each species, 10,000 background points were randomly generated within the study area (Elith et al. 2011; Hysen et al. 2022; Kass et al. 2021; Phillips et al. 2006).

### 2.7 Occurrence and Background Partitioning

Data were partitioned into ‘testing’ and ‘training’ data, at an approximate 1:1 ratio, using partitions = ‘checkerboard1’ in the ‘ENMevaluate’ function. Checkerboard1 is a variation of geographically structured partitioning which separates data into two bins based on simple spatial rules (Kass et al. 2021). The purpose of using a spatially explicit partitioning system is to limit the impact of spatial autocorrelation in both ‘testing’ and ‘training’ datasets (Legendre 1993; Radosavljevic and Anderson 2014). Spatially structured partitioning is preferred here, as random partitions can often lead to high levels of dependency between ‘training’ and cross‐validation datasets, inflating model performance statistics (Radosavljevic and Anderson 2014; Roberts et al. 2017).

### 2.8 Model Tuning and Selection

For each species, 15 models were generated, including each combination of feature classes (L, Q and LQ) and regularisation multiplier values (one to five). Two model selection procedures are suggested for MaxEnt modelling, AICc (Akiake's Information Criterion corrected for small sample sizes; Burnham et al. 2011) and a sequential technique which uses area under the curve (AUC) and omission rate at the tenth percentile (OR.10p) from cross‐validation to select the top model (Kass et al. 2021). Both model selection methods yielded highly similar top models, so we chose to present top models selected using the sequential method.

### 2.9 Independent Validation Data

To generate the independent ‘testing’ dataset, lizard surveys via either funnel‐ or pitfall‐trapping were conducted during the 2022/23 summer throughout the Mackenzie Basin. Gee's minnow traps (3 mm mesh; Tackle Factory, U.S.A.) were used at most sites to trap ground‐dwelling lizard species. Traps were positioned at random points within 19 different parcels of Public Conservation Land. Points were generated within the boundaries of each site using the ‘random points in a polygon’ function in QGIS (QGIS Development Team 2023). Traps were deployed as close to the Global Positioning System (GPS) point as possible, with allowances made for GPS inaccuracy, stability of deployment and flooding potential. Traps were baited with a piece of 2 cm^3^ canned pear (Pams pear quarters ‘in juice’; Whitaker, 1967). To prevent desiccation of interned lizards and invertebrates, we included a wet sponge (cellulose; 5 cm × 5 cm) in each trap. Sponges were cleaned with F10 disinfectant and rinsed thoroughly between deployments to prevent transfer of disease amongst lizards. Two layers of cover (grass, sticks and other debris) were provided inside each trap. The bottom layer covered the sponge and pear allowing lizards to hide within, important when considering the possibility of predation. The top layer was placed above the trap entrance on one side, providing shade for the trap. When on uneven ground, we built small ‘ramp’ structures to provide better access to the trap. Traps were deployed overnight, for no more than 20 h, before being collected. Interned lizards were removed and identified to a species level. Individuals were released into cover, as close to the trap site as possible.

Pitfall traps were used instead of funnel traps at four sampling locations where trap arrays were previously established. Pitfall traps were arranged into five lines, spaced 30 to 50 m apart, each consisting of 10 pitfalls. Individual traps were spaced 5 ± 1 m apart. Traps were baited and serviced as described for Gee's minnows, above. A pitfall trap consisted of a 4 L metal paint can set in the ground with a flat rock added to the bottom to provide refuge and limit predation risk. Traps were covered with wooden lids with ~2 cm spacers to provide shelter and to prevent larger animals from entering. Pitfalls were activated when the weather was suitable: high maximum temperatures (18°C–28°C), clear skies (providing direct sunlight), low rainfall and calm winds (consistently lower than Beaufort four). Pitfalls were run for four consecutive nights (5 days) and were checked each day, allowing for bait replacement, sponge re‐wetting and lizard processing. Processing and release of lizards was identical to that described for Gee's minnows.

Presence and absence data were collected from both trapping methods. Where a certain lizard species was captured, this was treated as a species presence, and *vice versa*. For example, a trap that caught only McCann's skinks would be treated as a presence point for that species and an absence location for all other target species. It is worth noting that absence values may not be true absences for the species, as lizards present in the surrounding area may not interact with trapping devices, more likely for the funnel traps that were in situ for shorter durations. However, most prediction rasters had multiple trapping devices within their extents, and trapping was carried out in conditions that favour lizard capture, limiting the risk of false absence. Unfortunately, little is known about the rate at which NZ lizard taxa are captured by pitfall or funnel traps, making it difficult to quantify any impact of differing detection rates. Southern Alps geckos are able to climb out of pitfall traps, particularly as the ambient temperature increases throughout the day (Hoare et al. 2007). Though we endeavoured to limit the impact of this phenomenon by checking traps early in the day, we still likely underestimate presence for this species.

### 2.10 Model Validation

Once the top model was selected, measures of model performance were assessed to inform each model's predictive accuracy. AUC values were calculated for each validation dataset and we also generated True Skill Statistics (TSS) for the independent (presence/absence) testing data. TSS provides a measure of a model's ability to discriminate between presences and absences at a specific threshold value and is largely independent of prevalence (Allouche et al. 2006), except at extreme prevalence values (Leroy et al. 2018; Somodi et al. 2017). TSS values were calculated using the threshold value with the greatest overall accuracy (maximum accuracy threshold), where the sum of sensitivity and specificity are maximised (Liu et al. 2013).

### 2.11 Null Models

To ensure that statistics from top models meaningfully represented model performance, we built null models for each species. Null models were built using localities that were randomly sampled from across the study extent. Models were then evaluated using the withheld data that were also used to test the real models (Bohl et al. 2019). Following the methodology outlined in Kass et al. (2020), for each model iteration, *n* locations were randomly sampled, where *n* is equal to the total number of real occurrences in the cross‐validation fold. Each model was then evaluated using the real occurrences in the withheld fold. Cross‐validation AUC was calculated for each null model and compared to the real model. This process was repeated 100 times for each species (Appendix 2).

## 3 Results

### 3.1 Top Models

Top models for each species varied notably; no regularisation multiplier or feature class was consistently selected (Table 2). OR.10p for Mackenzie, roamatimati and scree skink models were high (between 0.170 and 0.289), suggesting that models were overfit compared to cross‐validation testing data, and predictions may show a narrow indication of species' preferred habitat (Table 2). Overfitting was not obviously related to the number of coefficients in each model (between 12 and 19; Table 2). Few variables were included in all top models; however, water (Lake, Pond or River), croplands and forests had a ubiquitously negative impact (Table 3). All continuous variables were included in at least one model, whilst three categories of land use were not present in any top model. Common trends across species suggest that the assessed lizard species are less likely to be found in cropland, indigenous or exotic forests and more likely to occur in gravel habitats and in tall tussock grasslands (Table 3). Most species preferred more northerly aspects and locations with lower annual rainfall (Appendices 3, 4, 5, 6, 7, 8).

**TABLE 2:** Summary validation statistics for top models produced for each species of lizards, the common species, including grass (GRA) and McCann's (MCC) skinks, Southern Alps geckos (SAG) and the rare species, namely, Mackenzie (MAC), roamatimati (RMM) and scree (SCR) skinks. Presented are the feature class (the shape of predictor–response relationship; L, linear, Q, quadratic and LQ, linear and quadratic), regularisation multiplier (rm) and the number of coefficients for the top model (ncoef). Validation statistics include omission rates at the tenth percentile (OR.10), area under the receiver operator characteristic curve values for cross‐validation (AUC cross) and for independent data (AUC ind) and the True Skill Statistics (TSS) generated at the maximum accuracy threshold value (Thres.).

Species	fc	rm	ncoef	OR.10	AUC cross	AUC ind	Thres.	TSS
GRA	LQ	4	12	0.109	0.709 ± 0.010	0.528 ± 0.042	0.679	−0.204
MCC	Q	4	19	0.098	0.826 ± 0.002	0.638 ± 0.026	0.706	0.398
SAG	LQ	5	16	0.104	0.825 ± 0.006	0.570 ± 0.029	0.776	0.094
MAC	L	2	18	0.289	0.830 ± 0.015	0.829 ± 0.156	0.998	0.589
RMM	L	2	18	0.170	0.885 ± 0.002	0.596 ± 0.033	0.547	0.372
SCR	L	1	17	0.182	0.801 ± 0.029	0.955 ± 0.006	0.901	0.910

**TABLE 3:** Summary of variable coefficients from top models for each species of lizards, the common species, including grass (GRA) and McCann's (MCC) skinks, Southern Alps geckos (SAG) and the rare species, namely, Mackenzie (MAC), roamatimati (RMM) and scree (SCR) skinks. Coefficient values are shaded green for positive values and red for negative values. Quadratic coefficients (denoted by I) are not shaded.

	GRA	MCC	SAG	MAC	RMM	SCR
Predictor variable						
aspect_northness	0.055		0.221	0.717	0.926	0.668
slope_deg			0.078	−0.003	−0.064	0.033
temp_minColdMonth	0.202		−0.409		−0.036	−0.14
temp_maxWarmMonth			0.099	−0.137	−0.26	−0.004
solRad_meanAnn				2.721	0.113	
precip_ann	< −0.001		< −0.001	−0.001	−0.001	−0.001
I (aspect_northness^2^)		−0.167	−0.192			
I (temp_minColdMonth^2^)	0.017	0.011	−0.117			
I (temp_maxWarmMonth^2^)						
I (solRad_meanAnn^2^)	−0.001	−0.008	−0.018			
I (slope_deg^2^)	< −0.001	< −0.001	−0.002			
I (precip_ann^2^)		< −0.001				
LandUse						
1 anthropogenic infrastructure	0.128	−0.237		−1.053	−0.364	−0.516
10 sand, gravel, rock	0.089		0.403	0.182		1.705
14 permanent snow and ice		−0.158		−0.99	−2.459	
20 lake, pond, river	−0.474	−1.517	−0.109	−4.293	−3.465	−0.234
30 cropland	−0.996	−1.895	−0.227	−2.193	−5.584	−2.328
41 low producing grassland		0.155		−0.152	−0.275	−0.479
43 tall tussock grassland		0.157	0.328	0.657	0.281	
44 depleted grassland		−0.199	0.703		−0.24	0.216
45 herbaceous freshwater vegetation		−0.214		−1.29	−1.778	−1.701
50 scrubland		−0.614		−2.149	−0.998	−1.203
55 sub alpine shrubland		−0.336		−1.299		1.407
56 mixed exotic shrubland		−0.382		1.393	−0.032	−1.88
58 Matagouri or grey scrub	0.628	−0.27	0.267	−2.291	0.677	−0.397
64 forest	−0.969	−1.827	−0.854	−0.322	−4.37	−0.82

### 3.2 Predicted Suitable Habitat

**FIGURE 3:**
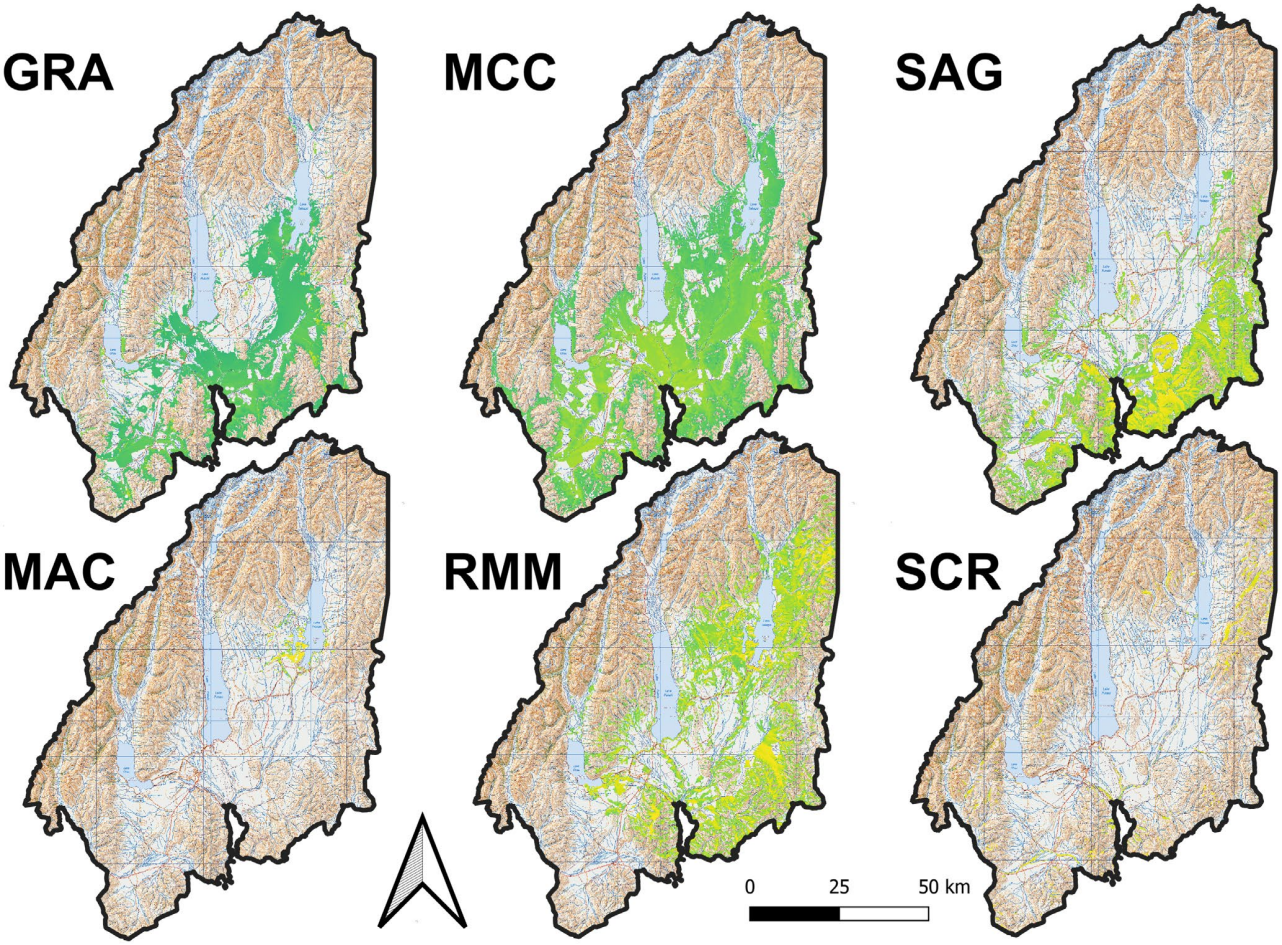
Predictive surfaces generated from the top model for each lizards species, including grass (GRA) and McCann's (MCC) skinks, Southern Alps geckos (SAG) and Mackenzie (MAC), roamatimati (RMM) and scree (SCR) skinks. Predictions are overlayed onto the Mackenzie Basin (New Zealand Topo250 series). Presented are predictions where values below the maximum accuracy threshold for each species are excluded.

Predictive surfaces representing suitable habitat were built for six lizard species throughout the Mackenzie Basin, each with a unique distribution. Grass skinks were predicted to occur mainly in the Basin lowlands, with lower occurrence probabilities at higher elevations and in marginal areas (e.g., lake and river side; Figure 3). The distribution of Mackenzie skinks centred around a single focal point near the southwest shores of Lake Tekapo (Figures 2 and 3). McCann's skinks were predicted to be widely distributed at the maximum accuracy threshold, present in both lowland and highland habitats (Figure 3). Similar to grass skinks, areas of high presence likelihood for McCann's skinks were often in marginal habitat. Predictions for roamatimati skinks did not extend to the southern portion of the basin (Figure 3). Roamatimati skinks were predicted to occupy high slope and elevation habitat, with low occurrence likelihood on the basin floor. Southern Alps geckos were predicted to be widely present in the hill country at the southeastern extent of the basin (Figure 3). Like roamatimati skinks, they were predicted to occur infrequently on the basin floor. All species were less likely to be present further west, into the Southern Alps.

### 3.3 Model Accuracy

**FIGURE 4:**
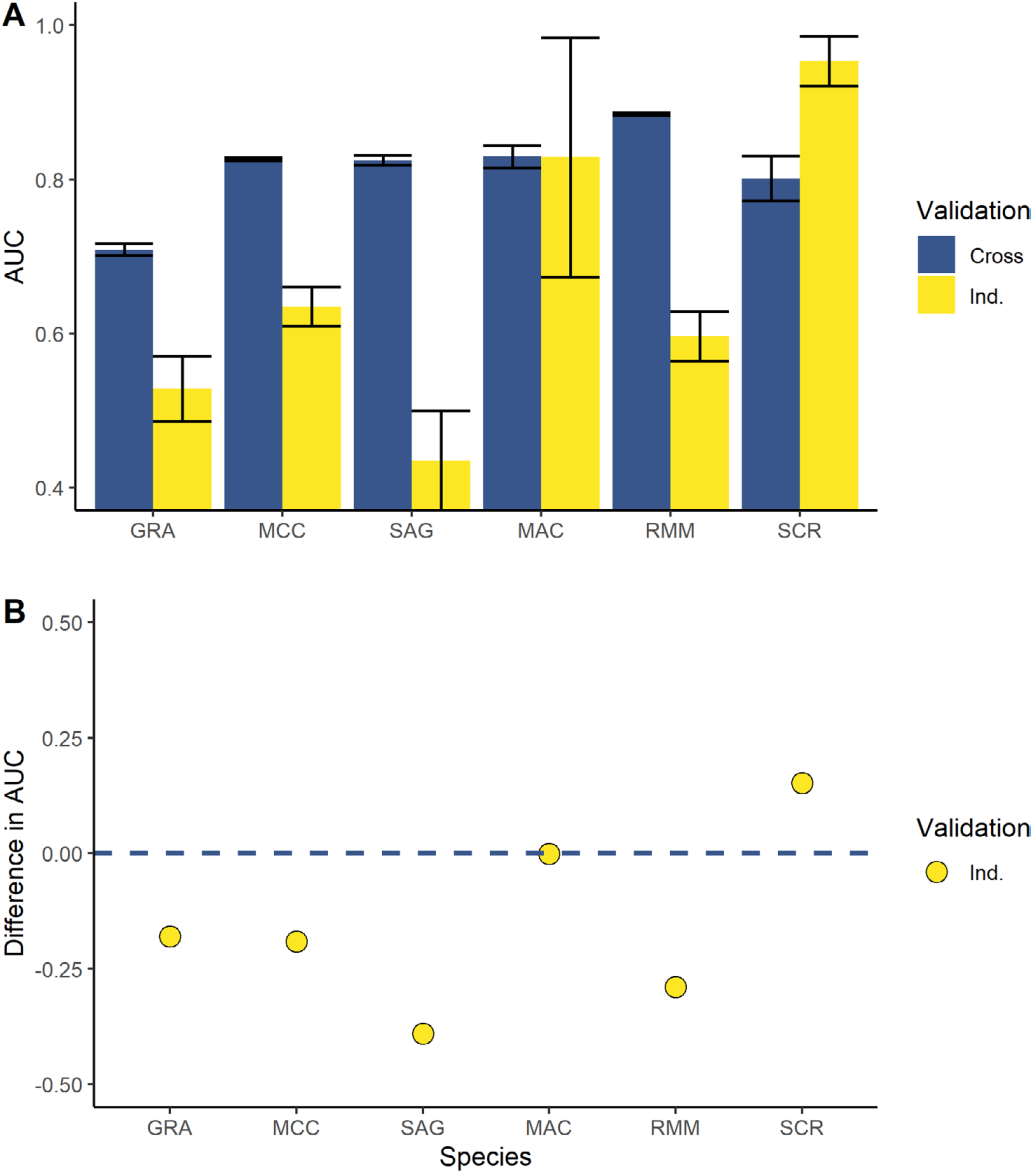
Area under the curve (AUC) values of top models when predicting model training cross‐validation (Cross) and independent datasets (Ind.). Values are displayed for each species of lizards, the common species, including grass (GRA) and McCann's (MCC) skinks, Southern Alps geckos (SAG) and the rare species, namely, Mackenzie (MAC), roamatimati (RMM) and scree (SCR) skinks. Displayed are AUC values (A) and the difference in AUC between cross and independent data (B).

Cross‐validation AUC values ranged from 0.709 to 0.885 (Table 1). Compared to cross‐validation results, models for common species performed notably worse when predicting to the independent testing data; AUC values were 0.181 (GRA), 0.188 (MCC) and 0.255 (SAG) lower. Models for rarer species performed variably when comparing to independent data; AUC values were equivocal (MAC), 0.154 (SCR) higher and 0.065 (RMM) lower (Figure 4 and Table 2). For independent testing data, TSS values correlated well with AUC values (Table 2). Top models for the common species had TSS values of −0.204 (GRA), 0.398 (MCC) and 0.094 (SAG). Mackenzie and scree skinks had TSS values above 0.5, indicating useful predictions for the independent data. Threshold values used to generate these values ranged widely, from permissive (0.547; RMM) to highly selective (0.998, MAC).

## 4 Discussion

Predictive modelling has the potential to improve sampling efficiency of rare lizard species. To be useful, model predictions must accurately describe the distribution of the target species. Here, we constructed SDMs with publicly available data and tested their utility for accurately predicting the distribution of three common and three rare lizard species across the Mackenzie Basin in NZ. The predictive abilities of models were tested using two distinct datasets: (1) cross‐validation data and (2) independent presence/absence data collected during the 2022/23 summer. For common species, cross‐validation results indicate that top models for each species generated reasonably accurate predictions; however, predictive accuracy decreased markedly when validating with independent data. For rare species, it was difficult to assess if predictions were accurate; both AUC and TSS values were high, but statistics were likely inflated by low sample sizes and spatial autocorrelation. These problems highlight limitations that will continue to hamper the creation and validation of models for such species in the foreseeable future.

In this study, cross‐validation indicated that predictive models for all species, bar the grass skink, were similarly performant. When predictive accuracy was tested with independent data, which likely gives a less biased and more temporally relevant indication of lizard distribution, model performance fell drastically for common species and roamatimati skink. Some level of decrease in predictive performance is unsurprising, given that validation data were collected in a restricted geographical area. Drivers of lizard distribution in the Mackenzie Basin may be different from those across the entire range of the species, limiting the predictive accuracy of models built with all available occurrence data (e.g., El‐Gabbas and Dormann 2018; Michael et al. 2010). We would expect this effect to be most pronounced for species that have limited sampling in the Mackenzie Basin, as location‐specific drivers would be underrepresented (e.g., grass skink), and for the model built with analogous species presence data as drivers likely vary amongst species. To limit the impact of this phenomenon it may be helpful to avoid using all occurrence data when building models, instead incorporating only geographically relevant occurrences (e.g., El‐Gabbas and Dormann 2018; Searcy and Shaffer 2014). However, limiting data in this way may result in models that are overfit to local or regional occurrences and fail to generalise well to unsampled areas. Further, limiting ‘training’ data is only feasible for species with high occurrence numbers (e.g., McCann's and grass skinks), which are not typically the primary targets of sampling and modelling efforts (Hitchmough et al. 2016). This problem highlights a paradox commonly faced when investigating the transferability of SDMs; predictions are most likely to be useful in areas without previous sampling effort, but predictions that don't account for location‐specific drivers are likely to be of lower accuracy (e.g., Michael et al. 2010; Zanini et al. 2009).

The decline in model performance could be explained by the quality of input data, lack of explanatory covariates and/or poor temporal or spatial relevance of included predictors (Fei and Yu 2016; Jarnevich et al. 2018; Rondinini et al. 2006; Seaborn et al. 2021; Sor et al. 2017; Syfert et al. 2013). The Herpetofauna Database contains occurrence data generated using diverse methodologies, including visual surveys, hand searching, pitfall and funnel trapping, artificial retreats and cover objects (Lettink and Monks 2016). The differences between how effort was applied for each method (e.g., random, systematic, incidental) coupled with altered detectability for each methodology, likely limits the comparability between measures of lizard presence/absence (Lettink and Monks 2016). Not applying effort equally in space, has been shown to inflate measures of model performance (Kramer‐Schadt et al. 2013; Veloz 2009). Whilst MaxEnt models are robust to some data inconsistency, the quality of presence data is still a primary determinant of model performance (e.g., Aubry et al. 2017; Crates et al. 2022; Leroy et al. 2018).

Often lizard habitat is defined on small scales, with the difference between ‘good’ and ‘bad’ habitat being separated by short distances. For example, Lettink and Seddon (2007) show that ground cover within a 1 m radius of a trap increases that devices' likelihood of capturing a lizard (McCann's and grass skinks), suggesting that lizards discriminate habitat on the scale of single scrub plants (also described by Chavel et al. 2012). In contrast, the land use variable used in this study and other available vegetation cover polygons or raster data only report the dominant vegetation types at broad resolutions (e.g., Newson 1987; Thompson et al. 2020). Variables at this scale are likely less relevant to lizard distribution than fine scale predictors, as they may ignore quality habitat. Unfortunately, for predictive models at the scale of the Mackenzie Basin (~5500 km^2^), fine scale predictor values are unavailable, and the discriminatory ability of said models likely suffers as a result. Whilst such data could be collected, time spent directly sampling for lizards in target areas would likely be a more effective use of limited conservation resources.

Several important drivers of lizard distribution are missing from the predictive modelling performed in this study. Principally, local predator abundances are known to heavily influence the ability of lizards to persist (Gasc et al. 2010; Murphy et al. 2004; Norbury et al. 2023; Reardon et al. 2012). Direct measurements of predator abundance/presence are unavailable across most of NZ's land area and useful proxies which describe mammalian predator distributions are limited. The inclusion of relevant predator measures is complicated by complex interspecific interaction amongst mammals, including mesopredator release and prey switching (Monks et al. 2024; Norbury et al. 2013; Smith et al. 2010). These factors make it difficult to know which predator groups (e.g., mustelids, rodents) are likely to impact lizards at which time (e.g., resource boom or bust). Even at low densities, predators can have significant impacts on lizards (e.g., Murphy et al. 2004; Norbury et al. 2023; Reardon et al. 2012), but may be undetectable by many conventional monitoring techniques (Pickerell et al. 2014; Smith and Weston 2017). Without a full understand of how mammalian predators and lizards interact it will be difficult to confidently include covariates that attempt to describe this relationship.

For Mackenzie and scree skink, independent validation indicated good model performance compared to other species. Improved performance is surprising given that the problems mentioned for common species are likely equally important or exacerbated for rare species. Unfortunately, the model statistics for rare species have several key detractors which increase uncertainty around how well they reflect reality. Principle amongst them are the high OR.10p values for all three models, 0.289 (MAC), 0.170 (RMM) and 0.182 (SCR). These high omission rates indicate that models are overfit and are unlikely to generalise well, which is important when attempting to apply models to unsampled areas. Overfitting can result when too many coefficients are included in models. This was a pertinent concern in this study where the number of levels of categorical variables were high (land use 15 levels). We do note that similar overfitting was not seen for common species despite similar numbers of coefficients in top models (Table 2). To investigate the impact of the categorical variables on overfitting we also modelled species occurrence without categorical variables (data not shown). In these simplified models, overfitting was marginally improved, though model performance for all species declined towards uselessness when validating with either testing datasets.

Small sample sizes likely affected the reliability of model statistics for Mackenzie and scree skinks, which each had fewer than 10 presence locations in independent testing data. As a result, correctly predicting few raster squares had a large impact on the accuracy of predictions, resulting in large, discrete changes to sensitivity. Low sample sizes could artificially inflate TSS values for these models, notable as only these two models performed well when validated with independent data. Another product of small sample sizes is the spatial autocorrelation of independent testing data which were not spatially trimmed to preserve the few occurrence data available for rare species (e.g., Mackenzie skinks). Consequently, many occurrences of rare species were tied to few individual raster squares (e.g., two raster squares for four scree skink occurrences). The most extreme example of this was for roamatimati skinks, where 112 occurrences fell within four raster squares, so validation statistics were driven by very few predictions. Such non‐independence limits the ability of validation metrics to explain how useful predictions are across larger geographic areas (Kramer‐Schadt et al. 2013; Legendre 1993; Veloz 2009). These problems indicate that presented results for rare species should be interpreted with caution, whilst also highlighting the issue of spatial scale for predictive surfaces and the difficulties of collecting sufficient distribution data to build and validate models.

Given the multitude of issues we highlight when building and validating predictive models for rare species, it is difficult to confidently assess whether or not they could be useful for increasing sampling efficiency or other applications. Importantly, the problems experienced in this study are unlikely to be unique to the focal species. Many of NZ's lizards are known from one or few populations (e.g., Sinbad skink; *O. pikitanga*, awakōpaka skink; *O. awakopaka*, white‐bellied skink; *O. hoparatea*), so may also provide few, highly correlated occurrences. For other taxa, the issue may be equally important, 16.1% of NZ's Threatened or At‐Risk invertebrate taxa trigger the ‘One Location’ qualifier in threat assessments, potentially describing limited occurrence records from a small number of similar locations (Buckley et al. 2015; Funnell et al. 2023; Heath et al. 2021; Hoare et al. 2017; Leschen et al. 2012; Mahlfeld et al. 2012; Rolfe et al. 2022; Sirvid et al. 2021; Trewick et al. 2022; Walker et al. 2022; Ward et al. 2014). Predictive models built for such species, are unlikely to generalise well to unsampled areas if trying to improve sampling efficiency (Radomski et al. 2022; Bennington et al. 2024). In addition, obtaining enough presence locations to independently validate predictive models for rare species may be too inefficient given limited resources. For example, we were unable to robustly independently validate models for rare species, despite over 1200 unique trap placements.

For NZ's lizards, it is unlikely that predictive surfaces would ever be the only factor considered when choosing sites to sample. Instead, model predictions would likely be combined with expert opinion and site‐specific requirements to prioritise areas for sampling, as suggested by Le Lay et al. (2010) and Costa et al. (2010). In this way, even models with limited predictive performance may be useful. Highlighted areas could be assessed by experts and decisions could be made using a synthesis of model predictions and opinion. In the future, improvements to modelling techniques, quality of input data (e.g., the use of LiDAR technologies to characterise habitat in higher resolution) and a greater knowledge of the drivers of species distribution may allow the improvement of models to a point where they provide powerful, highly accurate predictions of lizard distribution. However, we show that predictive surfaces generated using currently available data likely offer little utility for common lizard species in NZ. We also highlight key limitations of building and validating models for rare species. We caution taking cross‐validation at face value when using the NZ Herpetofauna database to build models as statistics may overestimate predictive accuracy, which is dangerous if predictions are used to inform sampling or management.

## Author Contributions

**Scott D. Bourke:** conceptualization (lead), data curation (lead), formal analysis (lead), investigation (lead), methodology (lead), writing – original draft (lead), writing – review and editing (lead). **Steph M. Bennington:** conceptualization (supporting), formal analysis (equal), methodology (equal), writing – review and editing (equal). **Sam Turner:** data curation (equal), writing – review and editing (equal). **Joanne M. Monks:** conceptualization (equal), formal analysis (supporting), funding acquisition (lead), investigation (supporting), methodology (equal), supervision (lead), writing – review and editing (equal).

## Conflicts of Interest

The authors declare no conflicts of interest.

## Data Availability

The data that support the findings of this study are available on GitHub at https://github.com/LemonL1meSuprIse/Evolution‐Ecology. New Zealand lizards are vulnerable to poaching; as such, available occurrence data has been jittered to limit any risk of it being misused. All environmental data are publicly accessible at https://datastore.landcareresearch.co.nz/dataset/nzenvds.

## Appendix 1

Names and number of land use categories that were joined together in order to reduce the number of model coefficients. Land use names are consistent with the New Zealand Land Cover Database (LCDB) classes at version 5.

**Table:**

Names of land use categories	Number	Collapsed number
Surface mine or dump, transport infrastructure, urban parkland/open space, built‐up area (settlement), not land	6, 5, 2, 1, 0	1
Manuka and/or Kanuka, Gorse and/or Broom, Fernland	52, 51, 50	50
Gravel or rock, landslide, sand or gravel	16, 12, 10	10
Estuarine open water, river, lake or pond	22, 21, 20	20
High producing exotic grassland, orchards, vineyards or other perennial crops, short‐rotation cropland	40, 33, 30	30
Exotic forest, indigenous forest, broadleaved indigenous hardwoods, forest‐harvested	71, 69, 64, 54	64

## Appendix 2

Summary of null models generated for each grass (GRA), Mackenzie (MAC), McCann's (MCC), roamatimati (RMM) and scree (SCR) skinks and Southern Alps geckos (SAG). One hundred null models were run for each species, binned by area under the curve (AUC) values. The blue line represents the cross‐validation AUC for the top model for each species.

## Appendix 3

Marginal response curves for the top model for grass skink, including predictors aspect_northness (−1 to 1), slope_deg (degrees), temp_maxWarmMonth (maximum temperature of the warmest month), temp_minColdMonth (coldest temperature of the coldest month respectively), annual solar radiation (solRad_meanAnn), annual precipitation (precip_ann) and land use. The *y*‐axis shows cloglog transformation values, an approximation of occurrence probability bounded by one and zero. Variables with horizontal curves or non‐varying bars are not included in the top model.

## Appendix 4

Marginal response curves for the top model for McCann's skink, including predictors aspect_northness (−1 to 1), slope_deg (degrees), temp_maxWarmMonth (maximum temperature of the warmest month), temp_minColdMonth (coldest temperature of the coldest month respectively), annual solar radiation (solRad_meanAnn), annual precipitation (precip_ann) and land use. The *y*‐axis shows cloglog transformation values, an approximation of occurrence probability bounded by one and zero. Variables with horizontal curves or non‐varying bars are not included in the top model.

## Appendix 5

Marginal response curves for the top model for Southern Alps gecko, including predictors aspect_northness (−1 to 1), slope_deg (degrees), temp_maxWarmMonth (maximum temperature of the warmest month), temp_minColdMonth (coldest temperature of the coldest month respectively), annual solar radiation (solRad_meanAnn), annual precipitation (precip_ann) and land use. The *y*‐axis shows cloglog transformation values, an approximation of occurrence probability bounded by one and zero. Variables with horizontal curves or non‐varying bars are not included in the top model.

## Appendix 6

Marginal response curves for the top model for Mackenzie skink, including predictors aspect_northness (−1 to 1), slope_deg (degrees), temp_maxWarmMonth (maximum temperature of the warmest month), temp_minColdMonth (coldest temperature of the coldest month respectively), annual solar radiation (solRad_meanAnn), annual precipitation (precip_ann) and land use. The *y*‐axis shows cloglog transformation values, an approximation of occurrence probability bounded by one and zero. Variables with horizontal curves or non‐varying bars are not included in the top model.

## Appendix 7

Marginal response curves for the top model for roamatimati skink, including predictors aspect_northness (−1 to 1), slope_deg (degrees), temp_maxWarmMonth (maximum temperature of the warmest month), temp_minColdMonth (coldest temperature of the coldest month respectively), annual solar radiation (solRad_meanAnn), annual precipitation (precip_ann) and land use. The *y*‐axis shows cloglog transformation values, an approximation of occurrence probability bounded by one and zero. Variables with horizontal curves or non‐varying bars are not included in the top model.

## Appendix 8

Marginal response curves for the top model for scree skink, including predictors aspect_northness (−1 to 1), slope_deg (degrees), temp_maxWarmMonth (maximum temperature of the warmest month), temp_minColdMonth (coldest temperature of the coldest month respectively), annual solar radiation (solRad_meanAnn), annual precipitation (precip_ann) and land use. The *y*‐axis shows cloglog transformation values, an approximation of occurrence probability bounded by one and zero. Variables with horizontal curves or non‐varying bars are not included in the top model.
